# Effects of COVID-19 Stress on Healing Behavior in Residential Spaces

**DOI:** 10.3390/bs13070524

**Published:** 2023-06-22

**Authors:** Jiyoung Oh, Heykyung Park

**Affiliations:** 1 Research Institute of Ecology, Pusan National University, Busan 46241, Republic of Korea; ojy1245@pusan.ac.kr; 2 Department of Interior Architecture, Inje University, Gimhae-si 50834, Republic of Korea

**Keywords:** COVID stress, healing behavior, residential space, healing environment

## Abstract

This study aims to analyze the effects of COVID-19 stress on healing behavior in residential spaces. Based on the results, the study further examines the residential space as a healing environment according to space use behavior in the post-COVID era. A survey including 2101 Korean people was conducted. The COVID Stress Scale for Korean People (CSSK) was used to identify COVID-19 stress. In addition, using a literature review on healing environment factors and behavioral changes after the pandemic, survey questions were developed to assess changes in healing behavior. A frequency analysis was conducted for sociodemographic factors, and the relationship between COVID stress factors and healing behavior factors in residential spaces was examined using factor and correlation analyses. Multiple regression analysis was conducted to verify the effects of COVID stress factors on changes in healing behavior. The results revealed that COVID stress affected healing behavior in residential spaces; however, there were differences in healing behavior depending on the COVID stress factors. Fear of being infected positively affected infection prevention behavior in homes. Anger towards others negatively affected the establishment of elements that support various activities in the residential spaces and furniture arrangement. Stress from social distancing difficulties affected healing behavior in residential spaces but was not related to infection prevention behavior. Residential spaces can serve as healing spaces when people are provided with various spatial factors that support diverse types of behavior during a pandemic crisis.

## 1. Introduction

The COVID-19 pandemic has caused dramatic changes in various parts of our lives. Since the pandemic, many scholars have studied various changes that have been caused by the pandemic [1]. One of the negative changes is an increase in stress levels and mental diseases, including depression [2,3,4,5] due to a deterioration in general health from a lack of physical activities [6,7] and a decline in social communication [8]. Several studies published after the outbreak of the pandemic focused on the negative changes in people’s physical and mental health [9,10].

Social distancing and quarantine policies to control the spread of the COVID-19 virus led to a great fear of being infected by the virus and limited the freedom of individuals [11]. Most quarantine occurred in residential areas which led to an increase in the amount of time spent there [12,13]. This caused residential spaces to become multipurpose locations for various functions, including work, learning, exercise, and leisure activities [14]. This led to negative results, including a decline in efficiency, productivity, and social exchanges [15], and caused rapid changes to people’s lifestyles and use of residential spaces [16].

Studies on residential space behavior published after the COVID-19 outbreak mostly focused on people’s diet, physical activity, sleep, and consumption behavior [17]. In detail, the longer the time spent in residential spaces, the more reports there were of unhealthy behavioral changes, including weight gain from a decrease in physical activities and increased food intake [18,19] and a decline in quality of life due to less sleep [13]. Studies indicated that increased physical activities led to lower stress levels, better quality of sleep, and other improvements in the overall quality of life and well-being [20]. Therefore, residential spaces serve as locations for physical activity and rest during quarantine. During the pandemic, the role of residential spaces expanded to include places to learn, work, exercise, and participate in leisure activities [21]. 

Geldart (2022) [22] stated that, to ensure safe and healthy working environments in residential spaces, personal working areas and ergonomic office equipment are needed, and it should be possible to boost self-regulation skills. Furthermore, a study that focused on changed consumption behavior during the pandemic found that, according to exigency motivation theory, there was a greater tendency to purchase economical items that satisfy basic needs rather than high-priced luxury goods [23]. There was an increase in purchases of pharmaceuticals, disinfectants, and other health and hygiene necessities, and consumption standards were reset to focus on price, availability, convenience, and hygiene [24]. As time spent in residential spaces increased during the pandemic, there was an increase in consumption patterns for items that allowed for engagement in various activities. For example, purchases of laptops and tablet PCs that enable working or learning from home [25], furniture that supports space to be used in various ways [26], and exercise equipment for working out at home increased; additionally, there was an increase in non-face-to-face purchases from online shopping malls [27]. Although several studies actively investigated various types of changes caused by the pandemic, most have focused on negative changes. It has been verified that stress due to the COVID-19 pandemic has harmed our society. 

One of the stressors caused by the COVID-19 pandemic is the fear of being infected. It has been verified that fear of being infected has positive correlations with preventive health behaviors [28]. Olapegba et al. (2022) [29] stated that the higher the fear of being infected, the more likely it is that people will participate in preventive health behaviors, including washing their hands or cleaning. However, it was also found that the psychological pain and stress that arise from the fear of being infected hinder preventive health behaviors. Fear of being infected positively affects preventive health behaviors, but psychological pain that comes from such fear can negatively affect health behaviors. Therefore, it was concluded that there is a need for appropriate education and health awareness campaigns to ensure that fear of being infected does not lead to psychological pain or stress.

Anger is another type of stress caused by the pandemic. During nationwide crises, including pandemics, people with negative feelings of anger are more likely to have lower awareness of risk and participate in risky behavior [30]. Specifically, people with high levels of anger are less likely to participate in social distancing measures or infection prevention activities led by the government [31]. However, there may be cultural differences regarding this issue. Choi and Kim (2022) [32] stated that, as it is characteristic of Korean culture to have a high awareness of community and to value compliance with social norms, Koreans are likely to abide by government policies despite experiencing stress from anger.

A possible stress factor from the COVID-19 pandemic is the difficulties that arise from social distancing or quarantine. It was found that social distancing and health behaviors are significant correlations; that is, people who engage in social distancing are likely to engage in health behaviors [33]. However, stress from difficulties resulting from social distancing leads to less participation in health behaviors and greater possibilities of stress-related diseases. Therefore, stress caused by social distancing, quarantine, and other government policies can negatively affect virus prevention behavior.

This part of the study focused on changes in individuals’ behavior and consumption patterns due to pandemic-related stress. When people are stressed, they engage in behaviors to relieve stress. This can manifest in the form of exercise, diet, and consumption patterns, as outlined in previous studies. However, during the pandemic, although people spent more time in residential spaces, it was difficult to find a case study that thoroughly investigated behavioral changes from the perspective of space planning. This study defined healing behavior as behavior undertaken to relieve stress. In summary, this study aimed to verify and investigate the effects of COVID-19 stress on healing behavior in residential spaces and focused on these spaces as a healing environment from a space planning perspective for the post-COVID era.

## 2. Materials and Methods

The study items are the COVID stress and healing behavior in the residential spaces of 2101 Koreans. The details of the study items and measurement scales are included in Section 2.1, Section 2.2, and Section 2.3 as follows.

### 2.1. Participants 

This study surveyed 2101 Koreans. The gender and age (20 s–60 s) of the survey participants were spread out as equally as possible, and the survey was conducted in March 2022. The number of reconfirmed cases of COVID-19 in Korea surged at that time, leading to stronger social distancing and quarantine policies, and the number of daily deaths was at its highest [34]. As it was difficult to conduct a face-to-face survey, participants were recruited through a professional online survey company. Among the 2101 responses, 43 that were considered inaccurate and irresponsible were excluded. Subsequently, 2058 responses were used for analysis in this study. All participants voluntarily participated in the survey, which was approved by the Pusan National University IRB (no. PNU IRB/2022_12_HR). 

### 2.2. COVID Stress Scale for Korean People (CSSK)

The COVID Stress Scale for Korean People (CSSK) is based on Taylor et al.’s (2020) tool and applied to the Korean environment to assess the COVID stress levels of Korean people [35]. Taylor et al. (2020) developed a COVID stress scale after evaluating 3479 Canadians and 3375 Americans based on the Patient Health Questionnaire-4 [36], the Short Health Anxiety Inventory [37], the Obsessive Compulsive Inventory—Revised [38], the Xenophobia Scale [39], and the Marlowe Crowne Social Desirability Scale Short Form [40,41]. The scale consists of 36 items. Among the items, those related to panic buying of food and xenophobia towards a certain race were not appropriate for the Korean people. Thus, Kim et al. (2021) [35] developed the CSSK, which was used in this study. The CSSK has an internal consistency of 0.955 with the tool developed by Taylor et al. (2020) [41] and consists of 20 items [35]. Each item is answered on a 5-point Likert scale. The effectiveness of the CSSK was verified by Kim et al. (2021) [42] and Park et al. (2022) [43], who used the tool in their studies.

### 2.3. Healing Behavior in Residential Spaces 

A survey with 23 items was developed to assess changes in healing behavior in residential spaces after the COVID-19 pandemic. All survey items were assessed using a 5-point Likert scale. The items were initially developed based on eight past studies on healing environment factors [44,45,46,47,48,49,50,51]. After developing the basic items, items that can assess changes in the healing behavior in residential spaces after the pandemic were developed based on four past studies related to post-pandemic lifestyle and behavior patterns [17,52,53,54].

(1) Healing Behavior

The concept of “healing behavior” that this study focuses on has not been mentioned in past studies. In this study, healing behavior refers to behavioral activities undertaken by space users to relieve stress. Specifically, it refers to stress-relieving behavior undertaken by using and controlling healing environment factors.

(2) Healing Environment Factors

Healing environment factors are those that compose a therapeutic and nurturing environment. According to Ulrich (1991), who first developed the concept, healing environment factors are environmental control, positive distraction, and social support [55]. This study analyzed eight past studies on healing environment factors and used the analysis to develop items that can assess healing behavior; see Table 1.

Controllability entails the provision of devices that can control temperature, sound, light, and humidity, and is discussed in all eight studies. In addition, natural factors that fall under positive distraction are related to providing natural items or space to view nature to promote healing. As most of the eight studies focused on medical spaces to investigate healing environment factors, it was difficult to apply accessibility and social support to residential spaces. Therefore, among the healing environment factors suggested by the eight studies, this study focused on controllability, safety, and positive distraction to develop a survey that can be applied to residential spaces. However, as this study aimed to focus on changes in the healing behavior in residential spaces during the COVID-19 pandemic, it was concluded that it is necessary to add details to the survey items by investigating past studies on behavioral changes that occurred due to the COVID-19 pandemic.

(3) Changes in the Use Behavior of Residential Spaces after the Pandemic

Four studies related to the changed residential space lifestyle or use behavior after the pandemic were reviewed. The studies were used as data to develop survey items that can identify changes in healing behavior in residential spaces after the pandemic began.

Kumari et al. (2020) [17] listed diet, physical activity, and sleep as lifestyle factors with the greatest changes after the pandemic broke out. To identify changes in lifestyle after the pandemic, Kumari et al. developed a survey with 20 items. Changes in diet were classified into food intake, cooking methods, and food consumption methods, while changes in physical activity were classified into exercise methods and participation in leisure and housework. Changes in sleep were classified into duration, quality, and stress and anger. It was determined that items related to cooking, exercise, leisure, and housework, which can be done within the homes, can be applied to the items for space factors.

Fabian Echegaray (2021) [52] focused on scenario-based sustainable lifestyle behavior from changes in consumption patterns and social relationships after the COVID-19 pandemic appeared. He proposed lifestyle behaviors of work, family, leisure, education, mobility, healthcare, food provision and consumption, and housing citizenship. Based on his findings, the items related to building an environment that supports various types of activities that occur in the home (work, leisure, education, healthcare, etc.) were developed. 

Kumari et al. (2020) [17] and Fabian Echegaray (2021) [52] focused on non-Koreans. It was concluded that their studies cannot be directly applied to Koreans because of cultural differences. Therefore, studies that investigated the changed residential use behavior of Korean people after the pandemic were used to develop a detailed set of items.

Kim and Jang (2021) [53] suggested that, as there is an increase in leisure and hobby-related activities that have occurred in residential areas after the pandemic, a convenient residential environment that incorporates IT is needed. As people work and study from home online, it is necessary to separate places of work and rest. Therefore, they suggested the use of versatile walls and other types of furniture. Moreover, as people spend additional time in residential spaces, the demand to improve personal space has increased, causing people to engage more in decorating their homes. 

Kang and Jang (2021) [54] analyzed the major types of living behavior of young one-person households using Internet news articles. The major types included hobbies (watching entertaining videos, taking online classes for self-development, crafts, home gardening), eating (delivery food, meal kits, drinking alone, desserts), shopping (purchasing food and other items related to housework), home decoration (learning about interior design trends, downloading interior design-related applications, etc.), and self-management (beauty, healthcare).

Based on the eight studies on healing environment factors and four studies on changed residential space use behavior after the pandemic, a survey with 23 items was finalized for this study. 

### 2.4. Statistical Analysis 

The sociodemographic characteristics of the participants were investigated using frequency analysis. The responses to the CSSK scale were examined using factor analysis to identify the COVID stress factors. In addition, the responses related to changes in healing behavior in residential spaces after the pandemic were analyzed using factor analysis to group common items into factors. Correlation analysis was conducted between the COVID stress factors and the changed healing behavior. Multiple regression analysis was used to verify the effects of COVID stress on healing behavior in residential spaces. Frequency analysis, factor analysis, correlation analysis, and multiple regression analysis were all conducted using the PASW Statistics 18 application.

## 3. Results

### 3.1. Sociodemographic Characteristics

A total of 2058 responses were used for the final analysis, and the sociodemographic characteristics of the participants are shown in Table 2. Their ages ranged from 20 to 60, and age and gender were equally distributed. There were approximately 400 participants from each age group and about 1000 participants from each gender. The most common monthly income of the participants was KRW3,000,000–KRW5,000,000 (30.4%) and KRW1,000,000–KRW3,000,000 (29.8%). Regarding educational level, 75.3% of the participants had a degree from a two-year college course or higher, indicating that most of the participants received post-secondary education. A total of 63.5% of Koreans live in apartments [56] and, in this study, the largest proportion of the participants (68.9%) also live in apartments. Participants who owned their homes accounted for 63.7%, followed by lease on deposit (chonsei) (21.3%), and monthly rent (13.6%). The percentage of the participants that lived in houses that are 100–132 m^2^ is 37.1%, followed by 32.5% who lived in houses with a size of 67–99 m^2^. In this study, 38.6% of the participants had been living in their current homes for one year or more to less than five years, followed by 21.1% with five years or more to less than 10 years.

### 3.2. COVID Stress Factor

The COVID stress of the participants was assessed using the COVID Stress Scale for Korean People (CSSK). Factor analysis was conducted to identify the COVID stress factors of the participants. The results are summarized in Table 3.

To extract these factors, the principal component extraction method and varimax rotation were used. All 20 items were found to be valid, therefore, factor analysis was performed. The KMO value was 0.931, and the *p*-value from Bartlett’s test was less than 0.000, indicating that the factor analysis model is appropriate. The cumulative variance was 62.667%, demonstrating that the three factors have strong explanatory power. The first factor was composed of eight items, and the second and third factors were composed of six items each. The first factor was related to anxiety and concern regarding infection and after-effects and was named “Fear of being infected”. The second factor was related to negative feelings towards those who did not comply with the quarantine guidelines and was named “Anger towards others”. The third factor, named “Difficulties from social distancing”, was related to the distinct types of depression, lack of social communication, and economic difficulties that arise from social distancing. The factor loadings were all 0.4 or above, satisfying the general validity levels of an assessment tool. Furthermore, according to an analysis of the reliability of the COVID stress factors, the reliability for all factors was 0.7 or above, demonstrating acceptable reliability levels. 

### 3.3. Healing Behavior Factors in Residential Spaces 

Factor analysis was conducted to identify the classification of healing behavior in residential spaces. The results are shown in Table 4. To extract these factors, the principal component extraction method and varimax rotation were used. Subsequently, aside from three items with low validity levels, a total of 20 items were used for the factor analysis. Three items were excluded from the analysis: “After the pandemic, I placed a fire extinguisher or a fire alarm at home as I was concerned about fire”; “After the pandemic, I purchased new cooking equipment to cook at home”; and “After the pandemic, I got a pet”.

After the factor analysis, it was found that the KMO value was 0.935, and the *p*-value from Bartlett’s test was less than 0.000, proving that the factor analysis model is appropriate. The cumulative variance was 62.239%, demonstrating that the five factors have strong explanatory power.

In terms of the items under each factor, the first factor was composed of eight items, the second factor comprised four items, the third and fourth factors were composed of three items each, and the fifth factor consisted of two items. The first factor was related to arranging new space elements to support various types of activities that can occur in the residential space, as people spend more time in their residential spaces due to the pandemic, and was named “Set up of elements that support various types of activities”. The second factor, named “Furniture arrangement for personal activities”, was related to rearranging furniture to secure personal space and ensure personal activities. The third factor, “Placing devices to control the quality of space”, was related to control that can organize or regulate the quality of space when people spend more time in their residential spaces. The fourth factor was related to behavior within residential spaces to prevent infection and was named “Hygiene activities to prevent infection.” The fifth factor was named “Placing natural elements to change the atmosphere.” All factors had factor loadings of 0.4 or above, satisfying the general validity levels of an assessment tool.

### 3.4. Correlation between COVID Stress and Healing Behavior in Residential Spaces 

Pearson’s correlation analysis was conducted to identify the correlation between major COVID stress factors and healing behavior in residential spaces. The results are shown in Table 5. The first COVID stress factor of “Fear of being infected” indicated significant positive correlations with “Set up of elements that support various types of activities (r = 0.069, *p* < 0.01)”; “Furniture arrangement for personal activities (r = 0.068, *p* < 0.01)”; “Placing devices to control the quality of space (r = 0.091, *p* < 0.000)”; “Hygiene activities to prevent infection (r = 0.255, *p* < 0.000)”; and “Placing natural elements to change the atmosphere (r = 0.105, *p* < 0.000)”. 

The second COVID stress factor of “Anger towards others” did not show a significant correlation with “Set up of elements that support various types of activities” and “Furniture arrangement for personal activities.” However, it revealed positive correlations with “Placing devices to control the quality of space (r = 0.089, *p* < 0.000)”; “Hygiene activities to prevent infection (r = 0.335, *p* < 0.000)”; and “Placing natural elements to change the atmosphere (r = 0.071, *p* < 0.01)”. 

The third COVID stress factor of “Difficulties from social distancing” showed high positive correlations with “Set up of elements that support various types of activities (r = 0.172, *p* < 0.000)”; “Furniture arrangement for personal activities (r = 0.180, *p* < 0.000)”; “Placing devices to control the quality of space (r = 0.119, *p* < 0.000)”; “Hygiene activities to prevent infection (r = 0.117, *p* < 0.000)”; and “Placing natural elements to change the atmosphere (r = 109, *p* < 0.000)”. 

### 3.5. Effects of COVID Stress on Healing Behavior in Residential Spaces 

Multiple linear regression analysis was conducted to verify the effects of COVID stress on health and healing behavior in residential spaces. The three COVID stress factors were independent variables, and the healing behavior factors in residential spaces were dependent variables. Table 6 displays the results of the analysis. The Durbin–Watson statistics all indicated a value close to 2, displaying no issues regarding the independence of the residuals. All VIF values were less than 10 and, thus, there were no issues regarding multicollinearity.

The regression model indicating the effects of COVID stress on the factor “Set up of elements that support various types of activities” was statistically significant (F = 25.430, *p* < 0.000). After verifying the significance of the regression coefficient, the “Fear of being infected” among COVID stress factors did not affect ”Set up of elements that support various types of activities” in residential spaces (β = 0.013), while “Anger towards others” negatively affected “Set up of elements that support various types of activities” (β = −0.085, *p* < 0.01). “Difficulties from social distancing” positively affected “Set up of elements that support various types of activities” (β = 0.190, *p* < 0.000).

The regression model showing the effects of COVID stress on the factor “Furniture arrangement for personal activities” was statistically significant (F = 24.760, *p* < 0.000). After verifying the significance of the regression coefficient, the “Fear of being infected” among COVID stress factors did not affect “Furniture arrangement for personal activities” (β = −0.008). “Anger towards others” negatively affected “Furniture arrangement for personal activities” (β = −0.050, *p* < 0.05). “Difficulties from social distancing” positively affected “Furniture arrangement for personal activities” (β = 0.198, *p* < 0.000).

The regression model demonstrating the effects of COVID stress on the factor “Placing devices to control the quality of space” was statistically significant (F = 12.349, *p* < 0.000). After verifying the significance of the regression coefficient, the “Fear of being infected” among COVID stress factors did not affect “Placing devices to control the quality of space” (β = 0.018). “Anger towards others” positively affected “Placing devices to control the quality of space” (β = 0.053, *p* < 0.05). Furthermore, “Difficulties from social distancing” positively affected “Placing devices to control the quality of space” (β = 0.095, *p* < 0.000).

The regression model displaying the effects of COVID stress on the factor “Hygiene activities to prevent infection” was statistically significant (F = 98.149, *p* < 0.000). After verifying the significance of the regression coefficient, the “Fear of being infected” (β = 0.140, *p* < 0.000) and “Anger towards others” (β = 0.279, *p* < 0.000), among COVID stress factors, positively affected “Hygiene activities to prevent infection”. “Difficulties from social distancing” did not affect the factor (β = −0.032).

The regression model illustrating the effects of COVID stress on the factor “Placing natural elements to change the atmosphere” was statistically significant (F = 10.871, *p* < 0.000). After verifying the significance of the regression coefficient, the “Fear of being infected” among COVID stress factors (β = 0.057, *p* < 0.05) significantly positively affected ”Placing natural elements to change the atmosphere”. “Anger towards others” (β = 0.024) did not affect” Placing natural elements to change the atmosphere”, while “Difficulties from social distancing” (β = 0.073, *p* < 0.01) significantly positively affected “Placing natural elements to change the atmosphere”.

## 4. Discussion 

This study aimed to identify the effects of COVID stress on changes in healing behavior in residential spaces and to investigate the residential environment from a space planning perspective for the post-COVID era. Studies related to changes in behavior after the COVID-19 pandemic have focused on physical activities, diet, sleep, and consumption. However, although people were spending more time in residential spaces after the pandemic, studies have not focused on the changes in behavior in homes because of COVID. The results of this study are now discussed.

This study was conducted in Korea in March 2022, and so reflects the COVID circumstances unique to Korea. In March 2022, Korea saw a resurgence in those who were confirmed multiple times and implemented stronger social distancing and quarantine measures. Therefore, most of the participants of this study had COVID stress, and so the findings of these studies cannot be the standard to interpret the results of studies conducted in other countries during the same time period. Although there have been a number of studies related to COVID, there has been no previous study regarding the healing behavior in residential spaces. A new finding from this study is that COVID stress affects healing behavior in residential spaces. In detail, fear of being infected, anger towards others, and difficulties that arise from social distancing practices particularly affect healing behavior in residential spaces. Accordingly, these behaviors can serve as an act of refuge to relieve COVID stress.

The three COVID stress factors identified in the study were “Fear of being infected”, “Anger towards others”, and “Difficulties from social distancing.” The COVID stress assessment tool used in this study was derived from Kim et al. (2021) [35]. The stress factors identified in this study were identical to the previous studies, thus supporting the findings of those studies. This shows that the CSSK assessing COVID stress demonstrates high reliability.

Healing behavior is a concept introduced by this study and refers to the space user’s behavior of controlling and using healing environment factors in a particular space. To conceptualize healing behavior in residential spaces, past studies that focused on healing environment factors were analyzed to categorize healing environment factors. Based on these findings, studies related to changes in behavior in residential spaces after the pandemic were analyzed to develop a survey that identified the healing behavior of participants. The changed healing behaviors in residential spaces after the pandemic were ”Set up of elements that support various types of activities”, “Furniture arrangement for personal activities”, “Placing devices to control the quality of space”, “Hygiene activities to prevent infection”, and “Placing natural elements to change the atmosphere”. 

“Fear of being infected” and “Difficulties from social distancing” had positive correlations with all five healing behavior factors of residential spaces. The effectiveness of each variable was examined with an additional analysis (multiple linear regression analysis).

“Fear of being infected” strongly affected infection prevention behaviors regarding hygiene and cleanliness in the residential space. This supports the findings of past studies [28,29]. “Fear of being infected” affects people so that they become more concerned with cleaning and ventilating the house, and purchasing cleaning equipment, hand sanitizers, and hand washes. People refrained from leaving the house because of the fear of being infected; they place natural elements (plants, etc.) in their homes to change or improve the atmosphere, which can lead to the alleviation of stress [57]. That is, the stress factor of “Fear of being infected” can positively affect people’s infection prevention behaviors by causing them to strictly manage their own space and personal hygiene even more. Therefore, when planning residential spaces with this aspect in mind, it may be helpful to create a place for people to wash their hands when coming indoors. Moreover, it may be helpful to provide a pantry to store cleaning equipment to aid in preventing infection. Furthermore, constructing modern windows and doors that can filter out fine dust and increase the levels of only clean air can help promote the health of residents. 

It was found that “Anger towards others” is likely to negatively affect “Set up of elements that support various types of activities” and “Furniture arrangement for personal activities”; that is, with greater anger towards others who do not comply with prevention measures, it is less likely that people will show healing behavior in homes. This partially supports the findings of past studies which revealed that anger leads to passive participation in activities that reduce the risk [29]. However, demonstrating the emotion of “anger” and how such an emotion is expressed may vary depending on cultural factors. According to previous studies, anger negatively affects healing behavior. However, it was found that anger positively affected “Placing devices to control the quality of space” and “Hygiene activities to prevent infection”. This may be related to the high sense of community of the Korean people, as mentioned in Choi and Kim (2022) [32]. Specifically, Koreans are more likely to follow personal hygiene measures and participate in the government’s infection prevention guidelines. If there is a wider range of control of the quality of space (sound, temperature, humidity, etc.) and its hygiene conditions, there will be less anger towards others, at least in one’s own home [58], contributing to relieving stress from COVID. 

As apartments are definitely the most common type of residence in Korea, setting up soundproof mats and windows that can effectively block sound and heat will help to control noise and temperature. Also, considering the climatic characteristics of Korea where humid conditions prevail in the summer and dry conditions prevail in the winter, it will be helpful to control such conditions by setting up dehumidifiers or humidifiers.

“Difficulties from social distancing” affected the various types of healing behavior in residential spaces. Those who experience difficulties caused by social distancing will most likely also experience a decrease in external activities, leading to considerable stress from the decrease in social communication. This is likely to occur among people who enjoy outdoor activities. Thus, these people will have a strong urge to satisfy their need to engage in the various outdoor activities that they participated in before the pandemic in their homes. They may want to satisfy their desire for outdoor activities by using their homes in distinct ways and placing various objects in their homes. This is why this factor affects “Set up of elements that support various types of activities”, “Furniture arrangement for personal activities”, “Placing devices to control the quality of space”, and “Placing natural elements to change the atmosphere”. 

Therefore, to establish elements to support various activities, people may search for various interior design factors and show greater interest in interior designs that can reflect their own taste against this backdrop. Additionally, there will be more sales of versatile furniture or storage furniture that can facilitate personal activities and protect privacy. People learned that it is important to secure personal areas in residential spaces to work or learn from experiencing the pandemic. Therefore, providing various interior design factors that can secure personal space in residential spaces can be helpful in reducing the stress resulting from the pandemic. There will probably be greater demand for electronics that can control the quality of space. Among the various natural elements, plants that purify air and pictures (or photographs) of natural landscapes can help people satisfy their desire for outdoor activities. However, the factor “Difficulties from social distancing” was shown to be inconsequential regarding infection prevention. This is in line with past studies that revealed that, when people have high stress levels because of adopting social distancing measures, they are less likely to participate in health-related activities. 

When reviewing the results of Table 6, three factors associated with COVID stress were found to be the most effective on “Hygiene activities to prevent infection” among healing behaviors in residential spaces. To prevent the spread of another pandemic in the post-COVID era, it will be necessary to formulate plans with regard to living spaces that can allow hygiene activities in the residential spaces. For example, when remodeling, it may be helpful to set up a sink to wash the hands near the entrance, install good quality ventilation systems, or purchase air purifiers. Using hand sanitizers and cleaning sanitizers in homes can also help to prevent new infectious diseases.

## 5. Conclusions

The findings of this study can be summarized as follows.

The changes in healing behavior of the Korean people in residential spaces were affected by COVID stress. In conclusion, the most important factor that should be given top priority to support healing behavior in residential spaces was found to be “Hygiene activities to prevent infection”.

However, factors related to healing behavior differed depending on the COVID stress factors. Among the stress factors, “Fear of being infected” affected infection prevention behaviors, including the management of cleanliness and hygiene in homes. “Anger towards others” negatively affected the placing of furniture and elements that allow for several types of activities in the homes, but this may produce different results depending on cultural context. Stress from “Difficulties from social distancing” affected the various healing behavior factors in residential spaces but did not affect infection prevention behaviors, including management of cleanliness and hygiene in the homes. As distinct COVID stress factors lead to various healing behaviors in residential spaces, it will be helpful to reduce people’s stress during a pandemic when residential spaces serve as healing spaces. Therefore, it is necessary to provide diverse environmental elements that support various types of behavioral changes from a space-planning perspective. 

## 6. Limitations and Future Study 

This study provides insights into understanding and identifying the effects of COVID stress on changes in healing behavior in residential spaces. However, this study had some limitations.

First, this study aimed to focus on the effects of COVID stress experienced by the Korean people as a collective unit regarding changes in healing behavior in residential spaces. Hence, analyses of sociodemographic and residential characteristics were not conducted. With a cross-analysis of multiple groups considering personal characteristics, it will be possible to provide more in-depth results on the direction of residential environment planning. This will be addressed in a follow-up study.

Second, the survey items used in this study were developed based on past studies on healing environment factors. In addition, as there were few studies on changed behavior in residential spaces after the pandemic, it was difficult to devise items on all types of healing behavior that can occur in residential spaces. It will be possible to provide more detailed results when developing items based on a qualitative study of actual residents, and when an expert evaluation, pilot evaluation, and other complex and detailed processes are undertaken.

Third, the explanatory power of R^2^ in the regression analysis was relatively low, which may have undermined the results of the study. However, aside from the R^2^ value, all other values that assess significance were shown to be appropriate, and although the R^2^ value was low, based on other studies that have conducted regression analysis [59,60,61], it was decided that it is possible to conclude even with a relatively low R^2^ value. Therefore, it is suggested that future research makes improvements to this study and increases the number of samples to obtain an R^2^ value with greater reliability. 

## Figures and Tables

**Table 1:** Analysis of Past Studies on Healing Environment Factors.

	Healing Environment Factors	Detailed Factors	Details	Reference							
				A	B	C	D	E	F	G	H
1	Controllability	Privacy	- Space structure and furniture arrangement that protects privacy				●		●	●	
		Control of quality of space	- Temperature: Air conditioning/heating, HVAC controlling device - Sound: Sound insulation devices (Doors, windows) - Light: Lights with different brightness levels that can be moved - Air: Air purification and ventilation devices, air freshener - Humidity: Humidifier, dehumidifier, and other humidity control devices	●	●	●	●	●	●	●	●
2	Safety	Hygiene	- Place to wash hands to prevent infection - Materials that can be easily cleaned	●	●		●	●	●		
		Accident prevention	- Floor and wall materials that can mitigate shock and prevent falls - Securing safety for corners of furniture and pillars - Fire alarms, emergency exits, fire extinguishers	●		●	●				
		Security	- Furniture with locks, CCTV, security systems				●				
3	Accessibility	Finding routes	- Clear sign systems - Strategic placement of landmarks and major features - (major features that help find location)	●			●	●	●	●	●
		Traffic flow	- Space arrangement and traffic flow plan that reduces movement - Space arrangement that can be intuitively understood				●	●	●	●	●
4	Positive distraction	Natural factors	- Indoor plants, indoor garden, water areas - Windows for lighting and views, furniture arrangement that allows people to sit near windows - External viewing area (balcony, terraces, etc.)	●	●	●	●	●	●	●	●
		Aesthetic factors	- Materials and colors that create a bright atmosphere - Placements of paintings, sculptures, and other works of art	●	●	●	●	●			●
5	Social support	Space that ensures interaction	- Space that allows for conversation with others (Lounge, day room, etc.) - Easily movable furniture		●		●		●	●	●
		Space for activities	- Space to play, take part in hobbies, engage in creative activities, exercise, work, etc.				●		●	●	●

Note. A: Huisman et al. (2012) [44]; B: Iyendo et al. (2016) [45]; C: Zhang et al. (2018) [46]; D: Sadek et al. (2020) [47]; E: Mourshed et al. (2012) [48]; F: Ulrich et al. (2004) [49]; G: Ulrich et al. (2008) [50]; H: Ulrich et al. (1992) [51].

**Table 2:** Sociodemographic Characteristics of the Participants.

Characteristics		N	Percentage
Age	20–29	399	19.4
	30–39	417	20.3
	40–49	408	19.8
	50–59	425	20.7
	60–69	409	19.9
Gender	Male	1021	49.6
	Female	1037	50.4
Monthly income	Less than KRW1,000,000	238	11.6
	KRW1,000,000 or more–less than KRW3,000,000	613	29.8
	KRW3,000,000 or more–less than KRW5,000,000	625	30.4
	KRW5,000,000 or more–less than KRW7,000,000	331	16.1
	KRW7,000,000 or more–less than KRW9,000,000	157	7.6
	KRW9,000,000or more	94	4.6
Educational level	Middle school graduate or under	14	1.2
	High school graduate	424	20.6
	2-year college graduate	306	14.9
	Bachelor’s degree	1066	51.8
	Master’s degree or higher	176	8.6
House type	Studio-style apartment	181	8.8
	Multi complex houses	249	12.1
	Apartments	1418	68.9
	Detached houses	194	9.4
	Others	16	0.8
Ownership type	Own house	1310	63.7
	Lease on deposit (chonsei)	439	21.3
	Monthly rent	279	13.6
	Others	30	1.5
Size	33 m^2^ or under	115	5.6
	34–66 m^2^	293	14.2
	67–99 m^2^	669	32.5
	100–132 m^2^	763	37.1
	133–165 m^2^	164	8.0
	166 m^2^ or over	54	2.6
Duration of residence	Less than 1 year	225	10.9
	1 year or more–less than 5 years	795	38.6
	5 years or more–less than 10 years	434	21.1
	10 years or more–less than 15 years	253	12.3
	15 years or more–less than 20 years	165	8.0
	20 years or more	186	9.0

**Table 3:** Analysis of COVID Stress Factors.

Factor Name	Factor Loadings	Eigen Values	Pooled Variance (%)	Cumulative Variance (%)	Reliability
COVID Stress Factor (CSF) 1: Fear of being infected					
I am anxious as I don’t know when and where I will get COVID.	0.843	5.525	27.625	27.625	0.935
I’m concerned that I will be very sick from COVID.	0.812				
I’m scared that there will be symptomless COVID patients near me.	0.812				
I am worried that my family members will get COVID.	0.793				
I’m worried that I may get COVID from an enclosed space that I often use (elevators, public transportation, etc.)	0.787				
I’m worried that I might get COVID and cause trouble for my family.	0.763				
I am scared of physical after-effects that may come even after getting better from COVID.	0.748				
I’m worried that someone else may get COVID from me.	0.727				
COVID Stress Factor (CSF) 2: Anger towards others					
I get angry when I see people going to high-risk facilities that can spread COVID (bars, clubs).	0.804	3.877	19.385	47.010	0.879
I get angry at people who talk with their friends or talk on the phone without a mask in an enclosed space.	0.804				
I am angry that I follow the quarantine rules but others do not.	0.779				
I get angry at people who do not quarantine properly.	0.758				
I get angry at my boss, superiors, and family elders who insist on meetups without considering the possibility of spreading COVID.	0.725				
I get angry at religious people who persist in face-to-face activities.	0.704				
COVID Stress Factor (CSF) 3: Difficulties from social distancing					
I am depressed that I cannot enjoy my hobbies or cultural events due to COVID.	0.775	3.131	15.657	62.667	0.811
I feel isolated from society as social distancing continues.	0.759				
As I spend more time at home due to COVID, I feel a loss of zest in life and feel lethargic.	0.709				
I fight more with my family due to COVID.	0.635				
I am faced with greater economic difficulties due to COVID.	0.627				
It’s hard for me to deal with the fact I cannot meet with my friends and family as much due to COVID.	0.570				
Varimax Rotation, KMO = 0.931, Bartlett’s x2 = 24,366.472 (*p* < 0.000)					

Note. strongly disagree (1)–strongly agree (5).

**Table 4:** Factor Analysis of Healing Behavior in Residential Spaces.

Factor Name	Factor Loadings	Eigen Values	Pooled Variance (%)	Cumulative Variance (%)	Reliability
Healing Behavioral Factor (HBF) 1: Set Up of Elements That Support Various Types of Activities					
To admire the nature outside, I set up furniture that I can sit on in the balcony or veranda.	0.727	4.131	20.654	20.654	0.880
I placed mats or rugs on the floor to reduce noise between floors.	0.707				
I set up CCTVs at the entrance to my house or in front of my house for security purposes, or other door locks or security systems (CAPS, etc.)	0.690				
I changed the space arrangement of my house for various types of activities, and made a room for exercise, hobbies, or play, etc.	0.680				
I bought exercise equipment to exercise at home.	0.668				
I purchased furniture that can be easily moved and used it for multiple purposes.	0.636				
I placed artwork including photographs, posters, or paintings to create a positive atmosphere.	0.599				
To control lighting, I changed the location of lights or bought new lights.	0.542				
Healing Behavioral Factor (HBF) 2: Furniture arrangement for personal activities					
For non-face-to-face activities (work from home, online classes, etc.), I bought new furniture or changed the furniture arrangement or space arrangement.	0.735	2.723	13.615	34.268	0.805
I changed the furniture arrangement to secure personal space.	0.710				
I bought new storage furniture to store personal items.	0.632				
I changed some part of the interior of my house (walls, flooring, curtains, etc.) to create a positive atmosphere.	0.627				
Healing Behavioral Factor (HBF) 3: Placing devices to control the quality of space					
I bought air conditioning or heating equipment including air conditioners, fans, heating, or electric pads to maintain an appropriate indoor temperature.	0.718	1.981	9.906	44.175	0.715
I bought a humidifier or dehumidifier to maintain an appropriate humidity level.	0.664				
I bought a laptop, tablet PC, or speaker to enjoy some alone time.	0.648				
Healing Behavioral Factor (HBF) 4: Hygiene activities to prevent infection					
I became more concerned with cleaning my house, cleanliness, and hygiene to prevent infection.	0.785	1.952	9.759	53.934	0.685
I bought hand sanitizers or hand soap to prevent infection.	0.774				
I ventilated my house more often to enhance the indoor air quality.	0.739				
Healing Behavioral Factor (HBF) 5: Placing natural elements to change the atmosphere					
I have plants in my house to create a positive atmosphere.	0.791	1.861	9.305	63.239	0.799
I bought an air purifier or air purifying plants to purify the air inside the house.	0.770				
Varimax Rotation, KMO = 0.935, Bartlett’s x2 = 17,324.649 (*p* < 0.000)					

Note. Strongly disagree (1)–strongly agree (5).

**Table 5:** Results of Correlation Analysis between COVID Stress Factors and Healing Behavior Factors.

Factors	CSF1	CSF2	CSF3	HBF1	HBF2	HBF3	HBF4	HBF5
CSF1	1							
CSF2	0.468 ***	1						
CSF3	0.503 ***	0.282 ***	1					
HBF1	0.069 **	−0.025	0.172 ***	1				
HBF2	0.068 **	0.002	0.180 ***	0.688 ***	1			
HBF3	0.091 ***	0.089 ***	0.119 ***	0.568 ***	0.575 ***	1		
HBF4	0.255 ***	0.335 ***	0.117 ***	0.210 ***	0.195 ***	0.332 ***	1	
HBF5	0.105 ***	0.071 **	0.109 ***	0.538 ***	0.495 ***	0.483 ***	0.327 ***	1

Note. ** *p* < 0.01, *** *p* < 0.001.

**Table 6:** Multiple Linear Regression Analysis of COVID Stress Factors on Healing Behavior in Residential Spaces.

Dependent Variable	Independent Variable	B	SE	β	t(p)	TOL	VIF
HBF1	CSF1	0.012	0.025	0.013	0.489 (0.625)	0.631	1.584
	CSF2	−0.087	0.025	−0.085	−3.469 ** (0.001)	0.779	1.285
	CSF3	0.201	0.027	0.190	7.552 *** (0.000)	0.744	1.345
	Adj.R^2^ = 0.034 F(p) = 25.430 (0.000 ***) Durbin–Watson = 1.981						
HBF2	CSF1	−0.008	0.026	−0.008	−0.301 (0.764)	0.631	1.584
	CSF2	−0.053	0.026	−0.050	−2.040 * (0.042)	0.779	1.285
	CSF3	0.216	0.027	0.198	7.873 *** (0.000)	0.744	1.345
	Adj.R^2^ = 0.033 F(p) = 24.760 (0.000 ***) Durbin–Watson = 2.037						
HBF3	CSF1	0.018	0.028	0.018	0.647 (0.518)	0.631	1.584
	CSF2	0.061	0.029	0.053	2.153 * (0.031)	0.779	1.285
	CSF3	0.113	0.030	0.095	3.760 *** (0.000)	0.744	1.345
	Adj.R^2^ = 0.016 F(p) = 12.349 (0.000 ***) Durbin–Watson = 1.926						
HBF4	CSF1	0.124	0.023	0.140	5.406 *** (0.000)	0.631	1.584
	CSF2	0.275	0.023	0.279	11.912 *** (0.000)	0.779	1.285
	CSF3	−0.033	0.024	−0.032	−1.338 (0.181)	0.744	1.345
	Adj.R^2^ = 0.124 F(p) = 98.149 (0.000 ***) Durbin–Watson = 1.959						
HBF5	CSF1	0.068	0.033	0.057	2.059 * (0.040)	0.631	1.584
	CSF2	0.032	0.033	0.024	0.963 (0.336)	0.779	1.285
	CSF3	0.102	0.035	0.073	2.892 ** (0.004)	0.744	1.345
	Adj.R^2^ = 0.014 F(p) = 10.871 (0.000 ***) Durbin–Watson = 1.998						

Note. * *p* < 0.05, ** *p* < 0.01, *** *p* < 0.001.

## Data Availability

No additional data available.
